# An optimized "hypoxia in a pill" regimen reverses neurodegenerative disease phenotypes in multiple preclinical models

**DOI:** 10.64898/2026.06.30.734089

**Published:** 2026-07-03

**Authors:** Hong Wang, Eizo Marutani, Luca Zazzeron, Marissa Menard, Laura Volpicelli-Daley, Fumito Ichinose, Vamsi K. Mootha

**Affiliations:** 1. Howard Hughes Medical Institute and Department of Molecular Biology, Massachusetts General Hospital, Boston, MA, USA; 2. Department of Systems Biology and Medicine, Harvard Medical School, Boston, MA, USA; 3. Broad Institute of MIT and Harvard, Cambridge, MA, USA; 4. Anesthesia Center for Critical Care Research of the Department of Anesthesia, Critical Care and Pain Medicine, Massachusetts General Hospital and Harvard Medical School, Boston, MA, USA; 5. Killion Center for Neurodegeneration and Experimental Therapeutics, Department of Neurology, University of Alabama at Birmingham, Birmingham, AL, USA

## Abstract

A growing body of pre-clinical research has demonstrated the therapeutic potential of chronic, continuous hypoxia (11% FIO2) for treating both rare and common forms of neurodegeneration (1). However, the chronic delivery of hypoxic gas poses both practical challenges and long-term safety concerns. We previously introduced a small molecule, “hypoxia-in-a-pill” regimen that combines the hemoglobin affinity enhancer (GBT440) -- which limits oxygen delivery to tissues -- with a HIF-2α inhibitor (PT2399) to prevent compensatory erythropoiesis that can be detrimental. While this regimen extended the lifespan of the *Ndufs4* KO mouse model of Leigh syndrome, its efficacy still did not match that of chronic 11% FIO2. Here we report an optimized combination that now utilizes GBT601, a second-generation hemoglobin affinity enhancer with longer half-life and greater hemoglobin occupancy, again with PT2399. Here we report that the GBT601/PT2399 combination achieved therapeutic hypoxia and demonstrated strong efficacy comparable to continuous breathing of 11% FIO2 by halting neurodegeneration and even reversing neurological symptoms in three different mouse models: Leigh syndrome, Friedreich’s ataxia, and Parkinson’s disease. The dual targeting regimen led to a striking extension in median lifespan in the Leigh syndrome model, from a median of ~62 day to 158 days, when initiated after onset of advanced disease. Importantly, body weight was stable with the combination and it did not induce any signs of pulmonary hypertension, likely due to attenuation of HIF-2α. Our findings motivate additional pre-clinical and even clinical studies to evaluate the safety and efficacy of the GBT601/PT2399 combination.

## Introduction

A growing body of research is demonstrating that chronic moderate hypoxia (CMH) can have widespread therapeutic benefits (1). Epidemiological studies have long suggested the benefits of living at high altitude. For instance, residents of Swiss (2) and Austrian (3) Alps and the western United States (4) exhibit lower mortality from cardiovascular diseases, stroke, cancer, and Alzheimer’s disease. Anecdotal evidence further suggests that high altitude may ameliorate motor symptoms in Parkinson’s disease (5). A notable “natural experiment” involving ~20,000 soldiers stationed at high altitude (3200-4800 m, equivalent to an inspired oxygen of 11-13% at sea level) for three years revealed a lower incidence of hypertension, type 2 diabetes, and ischemic heart disease compared to that of 130,700 peers stationed at sea level (6). While many other environmental variables such as climate, solar radiation, diet, and activity confound these human data, preclinical research has supported reduced oxygen as a candidate driver of these benefits. Preclinical mouse studies beginning in 2016 have demonstrated that continuous exposure to 7-11% oxygen provides profound protective effects across diverse models, including Leigh syndrome (LS) (7-10) Leigh-like syndrome (11), optic neuropathy (12), Friedreich’s ataxia (FA) (13, 14), multiple sclerosis (15), accelerated aging phenotype (16), recovery from myocardial infarction (17) and ischemic stroke (18, 19), and Parkinson’s disease (20). The therapeutic responses are evolutionarily conserved from worms to mice (10, 13). A number of mechanisms have been identified, including an attenuation of brain hyperoxia (9, 20), preservation of the forward activity of “fragile” mutant redox enzymes (10, 13), the restoration of iron-sulfur cluster activity (13), and a reduction in brain lipid oxygenation (20).

Crucially, capturing these benefits requires continuous rather than intermittent regimens. In the *Ndufs4* KO model of Leigh syndrome, daily 10-hour hypoxic bouts failed to provide benefit (8) while in the *shFxn* model of Friedreich’s ataxia, a 16-hour daily regimen proved detrimental (14). This failure likely stems from the elevated hematocrit and hemoglobin (Hb) induced by hypoxia, which, upon return to normoxia, creates a physiological state analogous to hyperoxia. In support of this thesis, we showed that pharmacologically inhibiting HIF-2α can attenuate the detrimental effects of intermittent hypoxia (14). Safely delivering chronic continuous hypoxia raises practical challenges. The sporting industry has created a variety of portable nitrogen generators, tents, and face masks, that allow athletes to train in environments simulating high altitude. We previously “repurposed” such equipment in a study of healthy volunteers to achieve normobaric hypoxia over 5 days that nadired at 11% oxygen that was safe and well-tolerated (21). However, long-term exposure to chronic, continuous hypoxia is associated with a number of long-term, detrimental side effects, including pulmonary hypertension (PH) and right ventricular hypertrophy (RVH) in humans (22, 23) and in mice (24). These risks are particularly pronounced during development where neonatal hypoxia interferes with lung airway and vascular development, with reduced long-term survival due to severe PH and RV failure (24, 25). Furthermore, long-term hypoxia and sustained HIF-2α signaling are associated with complications such as paraganglionomas and glomus tumors (26). These challenges underscore the need to be able to deliver hypoxia in a way that is both practical and safe, leading us to introduce “hypoxia in a pill,” the first small molecule cocktail for inducing therapeutic hypoxia (27).

Our small molecule approach was based on dual targeting of hemoglobin and HIF2-αusing two classes of FDA approved drugs. First, we used the hemoglobin affinity enhancer GBT440 to decrease oxygen offloading and thereby decrease tissue oxygen levels. The hemoglobin affinity enhancers were originally developed to treat sickle cell anemia; Linus Pauling showed in the 1940’s that oxygenated hemoglobin – in the “R” state – has a lesser tendency to sickle (28), and the hemoglobin affinity enhancers stabilize the “R” conformation (29, 30). However, the decrease in oxygen offloading achieved with GBT440 induces a compensatory erythropoietic response which serves to increase tissue oxygen delivery and therefore limits the effectiveness of GBT440 monotherapy (27). Hence, we combined GBT440 with the HIF-2α inhibitor PT2399, which has been approved for vHL null renal tumors, to blunt the compensatory erythropoietic response (27). We showed that treatment with the combination could extend the lifespan of the *Ndufs4* KO mouse model of Leigh syndrome and delay neurological disease (27). A subsequent study showed that daily administration of HypoxyStat, a structural analog of GBT440 could also extend lifespan in the *Ndufs4* KO mouse model (31), though it is a covalent binder administered at extremely high doses and in contrast to GBT440, was not advanced into human trials (29).

While our initial combination (27) provided proof-of-concept, it remained less effective than inhaled 11% oxygen, likely due to the short half-life and limited Hb occupancy by GBT440. Here, we sought to optimize the regimen using GBT601 (Osivelotor), a potent, second-generation, clinical-stage molecule (32-34). GBT601 exhibits ~4.8-fold greater exposure and a ~3.5-fold longer half-life in rats compared with GBT440 (32). In a murine model of sickle cell disease, GBT601 treatment resulted in both an increase in hemoglobin oxygen affinity and a reduction in sickling of red blood cells (RBCs) that exceeded the effects of GBT440 preclinically. These pharmacological improvements enable equivalent HbS occupancy at a lower dose, reducing treatment burden. Notably, GBT601 has already completed safety studies in healthy humans (33).

Here we sought to explore the therapeutic potential of the GBT601/PT2399 combination in preclinical mouse models of mitochondrial and neurodegenerative diseases. We show that this combination is tolerated without apparent adverse effects in wild-type mice, achieves durable systemic and brain hypoxia, and halts neurodegenerative disease in three distinct mouse models. Furthermore, when initiated during advanced disease, the regimen halts and even reverses existing neurological phenotypes in these models, demonstrating efficacy comparable to breathing 11% oxygen. Importantly, dual targeting of hemoglobin and HIF2-αachieves these results without the detrimental cardiac side effects typically associated with chronic hypoxia.

## Results

### Dual targeting of hemoglobin and HIF2-αinduces systemic hypoxia without apparent adverse effects

**Figure 1.**
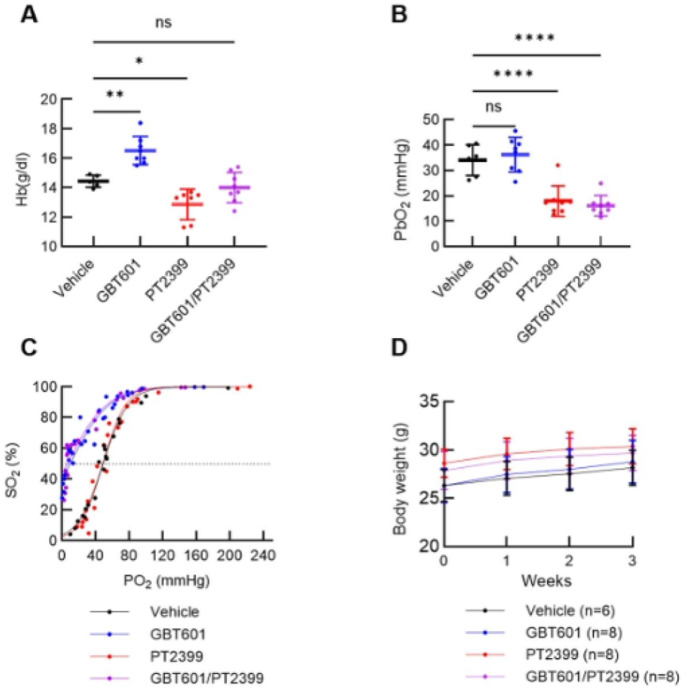
GBT601/PT2399 combination for induces systemic hypoxia. (**A**) Hb and (**B**) brain PbO_2_ measurements in vestibular nucleus of 10-week-old C57BL/6J male mice treated with vehicle, GBT601, PT2399 or the combination for 3 weeks (n=5-8 per group). (**C**) oxygen–hemoglobin dissociation curve measurements of 12-week-old C57BL/6J male mice treated with vehicle, GBT601, PT2399 or the combination for 7 days (n=4-5 per group). (**D**) Body weight of C57BL/6J mice treated with the indicated drugs. Bar plots show the mean ± SD. *P < 0.05, **P < 0.01, ***P < 0.001, ****P < 0.0001; 1-way ANOVA with Dunnett's test for multiple comparisons with vehicle, oxygen–hemoglobin dissociation curve compared by nonlinear regression (extra sum-of-squares F test).

We first measured effects of daily administration of GBT601 and PT2399 on hemoglobin levels and brain tissue oxygen partial pressure (PbO2) in wild-type (WT) C57BL6/J mice. We began by treating WT mice with each drug, individually or in combination, by oral gavage daily. After three weeks of daily treatment, the average hemoglobin in vehicle-treated mice (n=5) was 14.4 +/− 0.4 g/dL, whereas for mice treated with GBT601 alone it was 16.5 +/− 1.0 g/dL (n=8, P=0.002) and for those treated with PT2399 alone it was 12.9+/− 1.0 g/dL (n=7, P=0.02). The combination, however, led to Hb levels comparable to those with vehicle treatment (14.0 +/− 1.0 g/dL; n=8, P=0.8), confirming that the erythroid response to GBT601 is blunted by the HIF-2α inhibitor PT2399 (Figure 1A). Using an optical probe, we found that mice treated with GBT601 alone had similar PbO2 in the vestibular nucleus to the vehicle-treated (34.1+/− 6.1 mmHg vs. 36.2 +/− 6.8 mmHg, P=0.8), while those treated with PT2399 had significantly decreased PbO2 (17.9+/− 6.0 mmHg (P<0.0001 vs. vehicle). The combination of GBT601 and PT2399 decreased PbO2 to 16.1+/− 4.0 mmHg (P<0.0001 vs. vehicle) (Figure 1B).

Oxygen dissociation curves demonstrated distinct shifts among treatment groups. Vehicle-treated mice exhibited a typical sigmoidal oxyhemoglobin saturation curve with a P50 of 48.9 mmHg (95% CI: 47.1–50.7). PT2399 treatment alone did not substantially alter hemoglobin–oxygen affinity, with a P50 of 48.7 mmHg (95% CI: 45.7–51.9). In contrast, GBT601 treatment produced a marked left shift of the oxygen dissociation curve. The P50 was significantly reduced to 9.6 mmHg (95% CI: 5.8–12.9), reflecting increased hemoglobin–oxygen affinity and enhanced saturation at lower PO_2_ levels. The combination of PT2399 and GBT601 also demonstrated a pronounced leftward displacement, with a P50 of 6.4 mmHg (95% CI: 1.8–10.1), indicating preserved GBT601-mediated increases in oxygen affinity in the presence of PT2399 (Figure 1C).

Comparable to what we previously observed in mice treated with GBT440 and PT2399, the trajectory of bodyweight was the same for mice treated with vehicle, GBT601 alone, PT2399 alone and the combination of GBT601/PT2399. (Figure 1D). Thus, the combination of GBT601/PT2399 induces sustained brain tissue hypoxia and is well tolerated in wild-type mice for at least three weeks of continuous treatment.

### GBT601/PT2399 prolongs lifespan and reverses neurological phenotypes in Leigh syndrome mouse model

**Figure 2.**
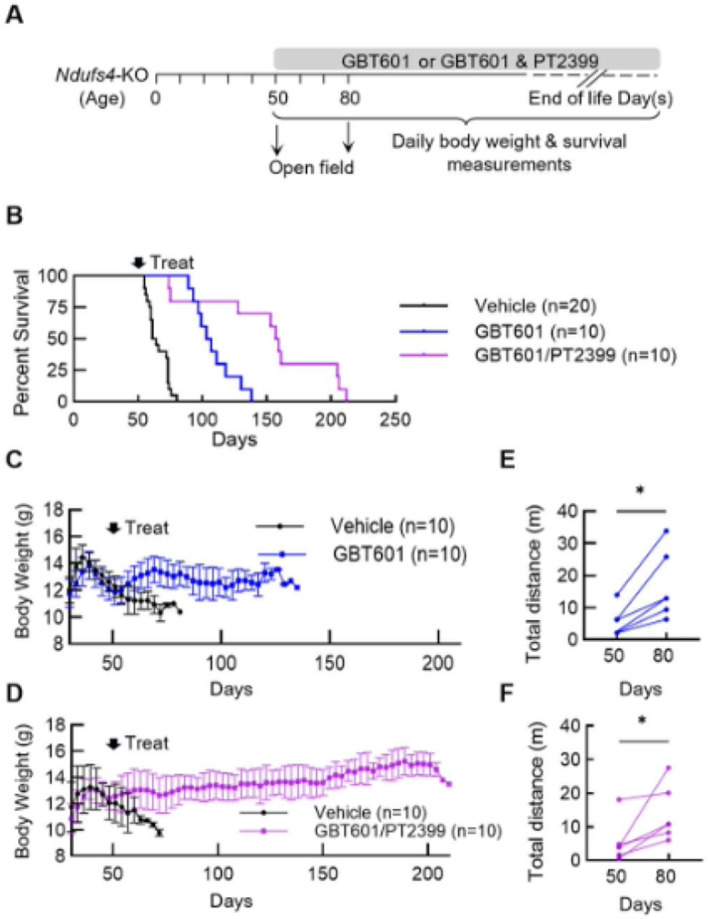
GBT601 alone or GBT601/PT2399 combination reverses neurological deficits and extends lifespan when initiated in advanced disease in *Ndufs4* KO model. **(A)** Schematic depicting the *Ndufs4*-KO mice experimental tests and time course. **(B)** Survival of *Ndufs4*-KO mice treated with vehicle, GBT601, or a combination. Body weight change of *Ndufs4*-KO mice treated with the GBT601 **(C)** or the combination **(D)**. Distance traveled in 15 minutes on an open-field test of *Ndufs4*-KO mice treated with GBT601 **(E)** or the GBT601/PT2399 combination **(F)** (n=6 per group at E, F). Bar plots show the mean ± SD. *P < 0.05, **P < 0.01, ***P < 0.001, ****P < 0.0001; paired t test; log-rank test for survival of drug-versus vehicle–treated mice. 2 way ANOVA test for body weight of drug- versus vehicle–treated mice.

Leigh syndrome, the most common pediatric manifestation of mitochondrial disease, is characterized by a subacute degeneration of deep gray matter regions and can be due to mutations in any of >110 different genes (35). We previously showed that chronic, continuous exposure to 11% FiO2 not only prevents pathology in the *Ndufs4* KO mouse model of Leigh syndrome (7), but when initiated in advanced disease, could halt further neurodegeneration and even reverse motor phenotypes (8). Moreover, we reported that GBT440/PT2399 could delay onset of disease and prolong lifespan by 30%, whereas neither drug was effective in isolation. Here we sought to evaluate whether the second generation GBT601 alone, or in combination with PT2399, are effective when initiated at day 50, when these mice exhibit very advanced neurological disease (Figure 2A). This is a more stringent approach (rescue versus prevention) but is more clinically relevant as most patients present after the onset of disease.

Ordinarily, *Ndufs4* KO mice are born healthy, show neurological defects on day ~35, begin to lose body weight, and succumb at ~2 months to their neurodegenerative disease. GBT601 alone increased median lifespan from ~62 to 105 days (P<0.0001) and maximum lifespan from 80 to 138 days (P<0.0001). Strikingly, the combination of GBT601/PT2399 extended median lifespan from ~62 to 158 days (P<0.0001) and maximum lifespan by from 80 to 212 days (P<0.0001, Figure 2B).

*Ndufs4* KO mice demonstrated an upward growth trajectory following initiation of treatment (Figure 2C-D). At day 70, when half of the vehicle groups had died, the average body weight of the GBT601 mice was higher than the surviving vehicle treated mice (13.4 ± 1.1g vs. 10.3 ± 0.6g, P<0.0001) (Figure 2C, S2A-B). Similarly, the body weight of the PT/GBT group was higher than the mice surviving in the corresponding vehicle group (12.6 ± 1.4 g vs. 9.8 ± 0.3 g, P<0.0001) (Figure 2D, S2C-D). Open field testing showed that distance traveled increased from day 50 to day 80 with either GBT601 (P=0.01, Figure 2E) or with the combination (P=0.048, Figure 2F).

### GBT601/PT2399 halts neurological disease phenotypes in Friedreich’s ataxia mouse model

Given the striking performance of the GBT601/PT2399 combination described above, we next evaluated its utility in a mouse model of Friedreich’s ataxia. Friedreich's ataxia (FRDA) is the most common inherited ataxia due to a recessive loss of the frataxin (FXN) protein (36-38). We previously reported that breathing chronic, continuous 11% oxygen improved ataxia in the doxycycline-induced *shFxn* mouse model (13), though hypoxia did not affect lifespan, as the cardiac pathology that is the proximal cause of death in this model is not ameliorated by hypoxia. We also previously reported that initiating chronic, continuous 11% FIO2 for 3 weeks can rapidly reverse features of ataxia when commenced 12 weeks after doxycycline-induced *shFxn*, at which point neurological debility is advanced (14).

**Figure 3.**
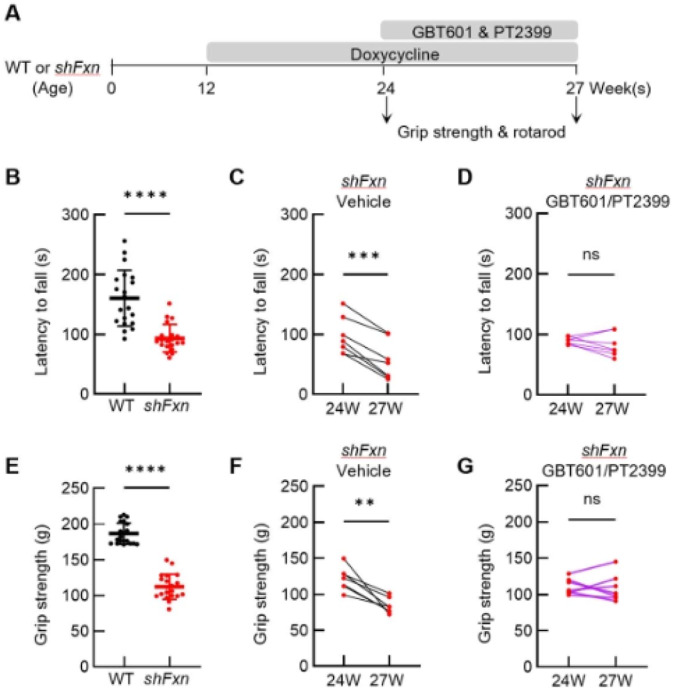
GBT601/PT2399 combination halts further ataxia in *shFxn* mice. **(A)** Schematic depicting the *shFxn* mice experimental tests and time course. (**B**) Rotarod test and (**E**) grip strength test in 24-week-old WT and *shFxn* mice treated with doxycycline for 12 weeks (n=20 per group at B, E). Rotarod test in *shFxn* mice treated with vehicle (**C**) or combination (**D**) at 24-week-old and 27-week-old. Grip strength test in *shFxn* mice treated with vehicle (**F**) or combination (**G**) at 24-week-old and 27-week-old (n=7 per group at C, D, F, G). Bar plots show the mean ± SD. n=group size. *P < 0.05, **P < 0.01, ***P < 0.001, ****P < 0.0001; paired *t* test for single comparison.

Here, we tested whether the GBT/PT combination could similarly halt or reverse neurological symptoms in the *shFxn* mice once they began to exhibit signs of advanced debility (Figure 3A). As expected (39), at 24 weeks of age (after 12 weeks of doxycycline) treatment, *shFxn* treated with doxycycline begin to exhibit decreased latency to fall on rotarod testing (Figure 3B). Between 24 weeks and 27 weeks, *shFxn* mice treated with the vehicle alone continue to exhibit deterioration, with their latency to fall on rotarod testing declining from a mean of 98 seconds to 57 seconds (paired T-test P=0.003, Figure 3C). In contrast, *shFxn* mice treated with GBT601/PT2399 preserved their time on the rotarod, with a mean latency to fall of 90 seconds at 24 weeks and 83 seconds at 27 weeks (paired T-test P=0.3, Figure 3D). At the 24 week time point (after 12 weeks of doxycycline treatment), sh*Fxn* mice exhibit marked decrease in grip strength (Figure 3E). Between 24 weeks and 27 weeks, grip strength continued to decline from a mean of 122 grams to a mean of 84 grams (paired T-test, P=0.003, Figure 3F), however, GBT601/PT2399 halted this decline, with grip strength being preserved from a mean 111 grams at 24 weeks versus mean of 109 grams at 27 weeks, (paired T-test, P=0.8, Figure 3G).

Hence, these studies indicate that initiating the combination treatment at 24 week of age, when sh*Fxn* mice exhibit advanced disease, can halt further progression of the ataxia phenotype.

### GBT601/PT2399 reverses neurological phenotypes in the α-syn PFF-induced mouse model of Parkinson’s disease

We sought to determine whether our small molecule drug combination might be effective in a mouse model of Parkinson’s disease (PD). We previously reported the therapeutic benefits of CMH in a α-syn preformed fibrils (PFF) mouse model of PD (20). In this model, PFFs induce endogenously expressed α-syn to form aggregates closely resembling Lewy Pathology in PD, leading to significant reduction of dopamine neurons in the substantia nigra pars compacta (SNpc) (40). Specifically, PFF-induced α-syn aggregation resulted in brain tissue hyperoxia, lipid peroxidation and dopaminergic (DA) neurodegeneration in the SNpc of mice breathing 21% oxygen. In mice breathing 11% oxygen, we found no difference in the abundance of α-syn aggregates, however, hypoxia attenuated the brain hyperoxia, lipid peroxidation, and DA neuron loss. Moreover, initiating hypoxia 6 weeks after PFF injection could reverse motor dysfunction and halted further DA neurodegeneration (20).

**Figure 4.**
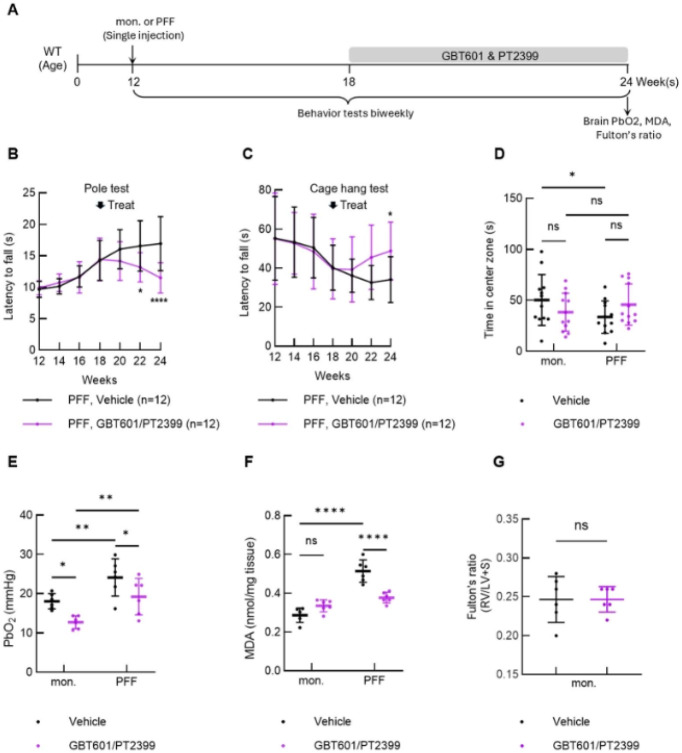
GBT601/PT2399 combination rescues α-syn-induced parkinsonism in mice. (**A**) Schematic depicting the mouse model of Parkingson’s disease experimental tests and time course. (**B**) Pole test and (**C**) Cage hang test in Parkinson's disease (PFF) mice treated with vehicle or the GBT601/PT2399 combination. (**D**) time in center zone on an open-field test of control (mon) and Parkinson's disease (PFF) mice treated with vehicle or the GBT601/PT2399 combination. (**E**) brain PbO_2_ measurements (**F**) MDA levels in SNpc tissues of control (mon) and PFF mice treated with vehicle or the GBT601/PT2399 combination. (**G**) Fulton’s ratio of control (mon) mice treated with vehicle or the GBT601/PT2399 combination. Bar plots show the mean ± SD. n=group size. n=12 per group at D. n=6 per group at E, F, G. *P < 0.05, **P < 0.01, ***P < 0.001, ****P < 0.0001. A two-way ANOVA for multiple comparisons was conducted.

We utilized a similar study design but now commenced with the GBT601/PT2399 drug combination (instead of inhalational hypoxia) after disease onset (Figure 4A). We administered intrastriatal injections of PFFs or monomeric α-syn as a control to 12-week- old mice to confirm onset of neurological disease at 18 weeks. We then treated the PFF mice with either GBT601/PT2399 combination regimen or vehicle from 18-week-old to 24-week-old and motor behavior was assessed using the pole test (coordination, bradykinesia), the cage hang test (coordination, balance), and the open field test (gross motor and anxiety-like behavior) (41).

Mice with injected with PFFs exhibit slowly increased latency to descend on pole testing from a mean of 9.7 seconds at 12 weeks to 14.3 seconds at 18 weeks, suggesting development of bradykinesia. Between 18 weeks and 24 weeks, mice treated with the vehicle alone continue to exhibit deterioration, with their latency to descend on pole testing, increasing from a mean of 14.3 secs at 18 weeks to 16.6 secs at 22 weeks to 16.9 secs at 24 weeks. In contrast, PFF-injected mice treated with GBT601/PT2399 decreased their latency to descend on the pole test, from a mean latency to descend of 14.4 secs at 18 weeks to 13.2 secs at 22 weeks (P=0.02, vs. vehicle), and 11.5 secs at 24 weeks (P<0.001, vs. vehicle, Figure 4B). Mice injected with PFFs showed marked impairment in cage hang testing. Between 12 weeks and 24 weeks, latency to fall in the cage hang test decreases from a mean of 55 seconds at 12 weeks to a mean of 40 seconds at 18 weeks. Between 18 weeks and 24 weeks, mice treated with the vehicle alone continue to exhibit deterioration, with their latency to fall on cage hang test decreasing from a mean of 40 seconds at 18 weeks to 34 seconds at 24 weeks. In contrast, PFF-injected mice treated with GBT601/PT2399 increased their mean latency to fall from 40 seconds at 18 weeks to 49 seconds at 24 weeks (P=0.03, vs. vehicle, Figure 4C). In open field testing, α-syn PFF treated mice showed decreased time in the inner zone (P=0.04) but the drug combination did not improve this phenotype (Figure 4D).

We previously reported that the α-syn PFF model exhibits brain hyperoxia and elevated levels of malondialdehyde, both of which were attenuated by chronic, continuous hypoxia breathing. We measured PbO2 in the SNpc 12 weeks after intrastriatal injection of PFF or monomer. Mice injected with PFF exhibited higher PbO2 (24.1 +/− 4.7 mmHg) compared to mice injected with α-syn monomer (18.1 +/− 1.9 mmHg, P<0.01) which was attenuated by the combination treatment (19.2 +/− 4.6 mmHg, Figure 4E). Mice with α-syn aggregates exhibited higher MDA levels 0.5 +/− 0.06 mol/mg compared to monomer-injected mice 0.3 +/− 0.04 mol/mg (P<0.0001). In contrast, the combination treatment attenuated the PFF-induced increase in MDA levels 0.4 +/− 0.03 mol/mg (P<0.0001 PFF vehicle vs. PFF GBT601/PT2399, Figure 4F).

Chronic hypoxia breathing is well known to drive pulmonary vascular remodeling in adults, including muscularization of small pulmonary arteries, increased pulmonary vascular resistance, and the development of pulmonary hypertension (PH) with consequent right-ventricular (RV) hypertrophy and, in severe cases, RV failure (22, 23). Fulton’s ratio—the weight of the right-ventricular free wall divided by the weight of the left ventricle plus septum—is a well-established marker of pulmonary hypertension. In mice, exposure to 11% hypoxia for 4–8 weeks typically increases this ratio from ~0.25 to ~0.35 (24). To determine whether the hypoxia in a pill drug combination therapy induced RV hypertrophy indicative of pulmonary hypertension (PH), we measured Fulton’s ratio in mice treated with vehicle or the GBT601/PT2399 combination for 6 weeks. Fulton’s ratio was similar in both groups (P=0.2, Figure 4G), suggesting that the drug combination did not induce PH.

Collectively, these studies in the α-syn PFF model of PD indicate that the GBT601/PT2399 combination drug combination reduces brain tissue hyperoxia and reverses neurological disease symptoms without any obvious toxic effects.

## Discussion

In the current report we have demonstrated that dual targeting of hemoglobin and HIF2-αwith the GBT601/PT2399 regimen is able to halt and even reverse disease in three models of neurodegenerative disease. An exciting aspect of our “hypoxia in a pill” combination is that it appears to be tolerated without any obvious adverse effects on body weight or cardiac remodeling. Our work motivates further evaluation of this drug combination both in pre-clinical disease models and in healthy humans.

It is notable that our small molecule-based, dual targeting approach - rationally designed to induce tissue hypoxia while preventing its compensatory erythropoietic response - closely parallels the genetic adaptations that naturally underwent selection in human populations living at high altitude who experience hypobaric hypoxia. The ascent to high altitude is associated with a compensatory, HIF-2α dependent erythropoiesis that strives to increase oxygen carrying capacity. This response is initially adaptive, but when prolonged can lead to detrimental side effects. Over evolutionary timescales, in certain populations such as the Tibetans, the chronic environmental pressure of high-altitude hypoxia has favored the selection of specific genetic variants in the EPAS1 (HIF-2α) pathway that blunt its activity (42-44). These adaptations prevent the maladaptive, excessive erythropoiesis typically seen in chronic mountain sickness, thereby protecting the cardiopulmonary system from the complications of hyper-viscosity. It is notable that in mice heterozygous loss of HIF-2α is protective against hypoxia induced pulmonary hypertension and right ventricular dysfunction (45). Our results demonstrate that this genetic mechanism observed in high altitude adapted human populations and in genetically engineered mice can be recapitulated pharmacologically with the HIF-2α inhibitor.

While the current work underscores the promise of a second-generation "hypoxia in a pill," significant safety and regulatory hurdles remain. Although both drug classes are FDA approved, the first-generation HbO2 stabilizer GBT440 (Voxelotor) has been voluntarily withdrawn from the market due to an excess of deaths attributed to vaso-occlusive crises (VOCs) (32). This drug was initially approved on the basis of a biomarker response, and post-approval surveillance revealed continued VOCs on the drug, which is currently under investigation. We note that the physiological consequences of increased hemoglobin oxygen affinity may differ substantially between sickle cell disease -- characterized by chronic anemia, microvascular dysfunction, and abnormal hemoglobin polymerization -- and a non-sickle context with intact oxygen delivery reserve. Here, we utilized GBT601, a follow-on compound with an improved half-life and higher hemoglobin occupancy that has demonstrated a favorable safety profile in preclinical models and Phase I human trials (33, 34). Nevertheless, elucidating the etiology of class-associated VOCs is essential, and “hypoxia in a pill” regimens may ultimately be contraindicated in patients with sickle cell disease (46). Furthermore, a key caveat remains regarding the use of HIF-2α inhibitors; while FDA-approved for certain renal cancers, they frequently induce anemia. Although we leverage this effect to counteract the polycythemia typically induced by enhanced hemoglobin affinity, the long-term consequences of this dual-pathway modulation must be carefully monitored. Further investigation will be vital to ensuring a responsible path toward confirming the safety and tolerability of this combination in humans.

## Materials and Methods

### Animals

All animal experimentation was approved by the Massachusetts General Hospital Institutional Animal Care and Use Committee. Mice had free access to food and water and were maintained in a 12-h light–dark cycle, at a temperature between 20 and 25 °C and humidity between 40% and 60%. The design of experiments involving animals followed the Animal Research: Reporting of In Vivo Experiments guidelines.

We used 10–12-week-old, age-matched and weight-matched male C57BL/6J mice (Jackson Laboratory). To minimize variability, we used a randomized paired (matched pairs) design.

*shFxn* mice were generously provided by the Geshwind Laboratory at the University of California, Los Angeles. Pups were weaned and genotyped at ~25 days after birth. Mouse genotypes from tail biopsies were determined using real time PCR with specific probes designed for each gene (Transnetyx, Cordova, TN). Body weights were recorded regularly, and mice were humanely euthanized when they had lost 20% of peak body weight, in accordance with the American Veterinary Medical Association guidelines. For all experiments, animals were randomized on a 1:1 basis, balanced by age and sex. The average age of the animals at the start of experiments was 3 months. Doxycycline treatment followed the established optimal dosing protocol (39); 2 mg/ml Doxycycline (Sigma) was added to the drinking water of all animals which was changed weekly. In addition, animals were injected intraperitoneally with doxycycline twice a week, starting with 5 mg/kg body weight for 10 weeks followed by 10 mg/kg body weight at later timepoints.

*Ndufs4* KO mice were generously provided by the Palmiter laboratory (University of Washington) (47, 48). Pups were genotyped and weaned at 21-28 d of age. Homozygous WT and heterozygous KO were used as littermate controls for *Ndufs4* KO mice as they appear identical in all assays performed by us and others. Natural death or 20% body weight loss (euthanasia criteria) were used to generate survival curves. For the *Ndufs4* KO mouse experiments, data from both sexes is combined as we found no differences between males and females, and no sex differences in disease phenotypes have been reported.

For PD mouse preparation, mouse α-syn synthesis and purification was performed at the University of Alabama at Birmingham as described previously (49). At 3 months of age, C57BL/6J mice were deeply anesthetized with isoflurane on a stereotaxic frame (MM 8000/3, ASI instruments). Animals were unilaterally injected with 2 μl of 300 μM sonicated fibrils, or 300 μM monomeric α-syn as a control, into the right striatum. Solutions were injected at a constant rate of 0.5 μl min^−1^ using a gastight syringe (cat#80030, Hamilton, Reno, NV) and a microinjection syringe pump (World Precision Instruments, Sarasota, FL) and the needle was left in place for 2 min; this was followed by slowly withdrawing the needle. The coordinates for the right striatum were +0.2 mm anteroposterior, ±2.0 mm mediolateral and −3.2 mm dorsoventral. All mice survived after stereotaxic injection of α-syn monomer or PFF and were used for the following experiment.

### Drug Administration

GBT601 and PT2399 were purchased from MedChemExpress. Mice were administered drugs by oral gavage daily: 100 mg/kg of the HIF-2α inhibitor PT2399 and 150 mg/kg of the GBT-601. Vehicle-treated mice were administered the vehicle for PT2399 (10% Ethanol, 0.25% Methyl cellulose,) and the vehicle for GBT-601 (10% DMSO, 0.25% Methyl Cellulose).

### Hemoglobin and hematocrit measurements

Eighty microliters of blood were collected by tail snip into a heparinized capillary. Hemoglobin concentration, hematocrit, and the saturation of hemoglobin with oxygen (O2Hb) were measured using a blood gas analyzer (ABL800 FLEX, Radiometer, Copenhagen, Denmark).

### Brain tissue PO_2_ measurement

For PbO_2_ in vestibular nucleus, mice were anesthetized with isoflurane (induction at 2–4%, maintenance at 1.5%), intubated, and mechanically ventilated with a tidal volume of 8 ml/kg, a respiratory rate of 110 breaths paer minute and an inspired fraction of oxygen (FiO_2_) of 21%. For PbO_2_ in SNpc, mice were anesthetized with isoflurane (induction at 2–4%, maintenance at 1.5%) with FiO_2_ of 21% via a nose cone. Mice were placed in a prone position and the head was stabilized using a stereotaxic frame (ASI Instruments, MI). After incision and dissection of the skin, an opening in the skull was performed using a micro-drill (MD-1200, Braintree Scientific, MA) and a PO_2_ probe was inserted at the desired location. The coordinates were ML = −1.25 mm, AP = −6.00 mm, and DV = −3.90 mm or ML = 1.20 mm, AP = −3.20 mm and DV = −4.40 from the bregma for vestibular nucleus or SNpc, respectively. Optical PO_2_ probe (OxyLab, Oxford Optronix, Abingdon, UK) was employed to detect the brain tissue PO_2_. During the brain PO_2_ measurement the depth of anesthesia was reduced by lowering the Isoflurane concentration to 1% to minimize the impact of anesthesia on the brain PO_2_.

### Oxygen Dissociation Curve Determination

Twelve-week-old mice received daily oral gavage with vehicle, PT2399, GBT601, or the combination of PT2399 and GBT601 for 7 consecutive days (n = 4-5 mice per group). Two to three hours after the last administration, mice were anesthetized with isoflurane, underwent tracheostomy, and were mechanically ventilated. The right carotid artery and right jugular vein were cannulated with PE-10 catheters for arterial and venous blood sampling, respectively. To generate oxygen dissociation curves, inspired oxygen fraction (FiO_2_) was stepwise adjusted to achieve a broad range of arterial and venous oxygen tensions (PaO_2_). At each FiO_2_ level, arterial and/or venous blood gases were obtained. Blood samples were analyzed immediately using a blood gas analyzer (ABL90 FLEX PLUS Radiometer). Oxygen saturation values used for curve construction were calculated as functional hemoglobin oxygen saturation (SO_2_) to account only for hemoglobin species capable of binding oxygen. Functional saturation was calculated as:

$$SO_{2}=\frac{O_{2}Hb}{O_{2}Hb+HHb}$$

Where HHb represents deoxyhemoglobin and is calculated as: 100−O2Hb−COHb−MetHb. Oxygen dissociation curves were constructed by plotting functional SO_2_ against corresponding PaO_2_ values. Data were analyzed using GraphPad Prism. Curves were fitted by nonlinear regression using a four-parameter logistic equation (log[agonist] vs. response). The upper and lower plateaus were constrained to 100% and 0% saturation, respectively, while P50 and Hill slope were allowed to vary freely.

### Accelerating rotarod measurements

A rotarod machine (Ugo Basile) was used to measure the ability of mice to stay on an accelerating, rotating rod. Mice were acclimated to the experimental room for at least 30 mins before the start of the measurements. Rotarod parameters were as follows: acceleration of 5 rpm/m and a maximum speed of 40 rpm. On each measurement day, three trials were performed, with individual trials at least 10 m apart to allow mice to recuperate. The median time on rotarod is reported. If mice used their body to grasp the rod (rather than walking on it) for more than 10 s, this time was recorded as time of fall.

### Grip strength test

fore and hind limbs grip strength was measured using a commercial dynamometer (Bioseb) over 3 consecutive efforts with 30 seconds of rest in between. The maximal effort (g) was used as absolute force (g) to assess whole-body strength. Mice were excluded if they refused the test (hanging < 10 seconds on 3 repeated attempts). Each mouse was tested three times, and the trials were averaged.

### Open Field Test

The apparatus consisted of four open-field boxes measuring 50x50 cm. Each box was surrounded by opaque black walls, and the whole apparatus was shielded from outside influences by walls with a height of 1.5 meters. After an acclimation period of 60 minutes inside the procedure room, one mouse was placed into the center of an openfield box. Up to four mice were tested in the apparatus simultaneously. The animals were allowed to explore the field freely for 15 minutes while being recorded by the ANYaze software with an overhead camera (v7.3, Stoelting Co., Wood Dale, IL). The position and movement of the animals were automatically tracked and analyzed by the ANY-maze software. The experimenters were blind to the treatment groups and, after starting the recording, left the room for the duration of the test. Before and after each test run, 70% ethanol was used to clean the apparatus.

### Pole test and cage hang test

Behavioral tests were conducted between 8:00 and 12:00 for the pole test and the cage hang test or 12:00 and 17:00 for the OFT. To conduct the pole test, a vertical metal pole with 2-cm diameter and 40-cm length was placed on the floor of a housing cage. Mice were placed on the top of the vertical metal pole and the time needed to reach the floor was measured. To conduct the cage hang test, a metal wire frame of the housing cage (WBL7115SMD-AMG, Allentown) was placed approximately 40 cm above the cage floor. Mice were hung on the metal wire frame and the time needed to drop onto the cage floor was measured. Each test was conducted five times and the average of the last three trials was used or the analysis.

### Detection of MDA

We measured the concentration of MDA in the SN tissue to evaluate the effect of drugs using a commercially available kit (cat. no. ab118970, Abcam). The assay was conducted according to the manufacturer’s protocol.

### Fulton's Ratio Measurement

After euthanasia, mouse hearts were excised from the thoracic cavities, the atria removed, and the free wall of the RV was separated from the left ventricle and the septum. The ratio of the weight of the RV to the weight of the left ventricle and the septum was calculated (Fulton's ratio). All measurements were performed by an investigator blinded to the treatment groups.

### Quantification and statistical analysis

Data are reported as mean±SD. Analyses were performed using GraphPad Prism 10.6.1 software. Two-way ANOVA with Bonferroni’s correction was used for multiple comparisons. 1-way ANOVA with Dunnett’s test for multiple comparisons with vehicle. Comparisons between 2 groups were conducted using the unpaired or paired Student's *t* test, as appropriate. P-value < 0.05 was considered to indicate statistical significance. *P < 0.05, **P < 0.01, ***P < 0.001, ****P < 0.0001; A log-rank test for survival of drug-versus vehicle–treated mice. oxygen–hemoglobin dissociation curve compared by nonlinear regression (extra sum-of-squares F test).

## Supplementary Material

Supplement 1
