# A second-generation ″hypoxia in a pill″ rescues neurodegenerative phenotypes across distinct mouse models

**DOI:** 10.64898/2026.06.30.734089

**Published:** 2026-09-04

**Authors:** Hong Wang, Eizo Marutani, Luca Zazzeron, Marissa Menard, Laura Volpicelli-Daley, Fumito Ichinose, Vamsi K Mootha

## Abstract

A growing body of pre-clinical research is demonstrating the therapeutic potential of chronic, continuous hypoxia (11% FIO2) for rare and common diseases (1). However, the chronic delivery of hypoxic gas poses both practical challenges and long-term safety concerns. We previously introduced a small-molecule, ″hypoxia-in-a-pill″ combining a hemoglobin affinity enhancer (GBT440) to limit oxygen delivery with a HIF-2α inhibitor (PT2399) to prevent detrimental compensatory erythropoiesis (2). Although this small-molecule regimen extended the lifespan of the Ndufs4 KO mouse model of mitochondrial complex I deficiency and Leigh syndrome, its efficacy did not match chronic 11% FIO2. Here we optimize this regimen using GBT601, a second-generation hemoglobin affinity enhancer with longer half-life and greater hemoglobin occupancy, and show it rescues key neurodegenerative phenotypes in multiple models. When we initiate therapy in 50 day old Ndufs4 KO mice with advanced disease, GBT601 monotherapy alleviated neurological disease phenotypes and extended median lifespan from 62 days to 105 days, while dual therapy with the GBT601/PT2399 combination extended median lifespan to 158 days. When initiated after onset of advanced disease in a mouse model of Friedreich′s ataxia, the combination halted further progression of motor phenotypes. In a mouse model of Parkinson′s disease due to a-synuclein toxicity, initiating the GBT601/PT2399 combination after onset of neurodegeneration attenuates brain hyperoxia and lipid peroxidation and reverses motor phenotypes. Importantly, the combination maintained body weight without inducing any signs of pulmonary hypertension. Our findings motivate further pre-clinical and clinical evaluation of our ″hypoxia in a pill″ approach for diseases with high unmet need.

## Full Text Availability

The license terms selected by the author(s) for this preprint version do not permit archiving in PMC. The full text is available from the preprint server.

