# Supplementary figures and images for: An optimized "hypoxia in a pill" regimen reverses neurodegenerative disease phenotypes in multiple preclinical models
Source: bioRxiv. 2026 Jul 3:2026.06.30.734089. Preprint. [Version 1] doi: 10.64898/2026.06.30.734089 (PMC13345306; doi:10.64898/2026.06.30.734089)

Body Weight (g)

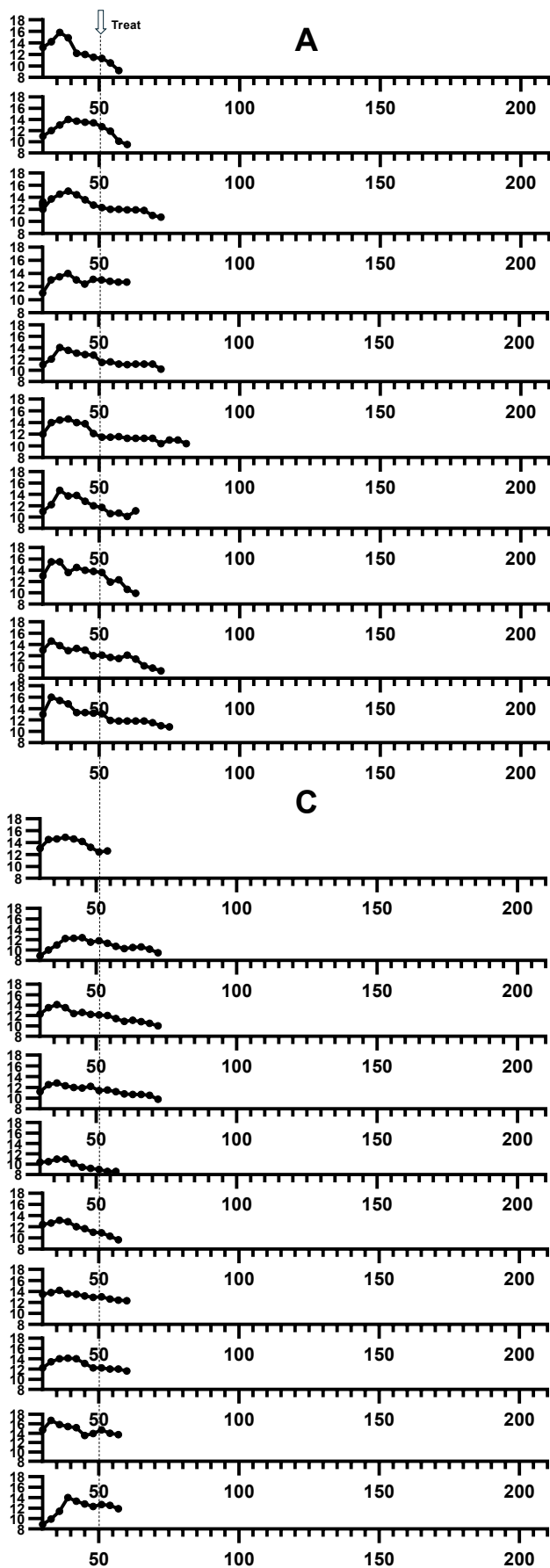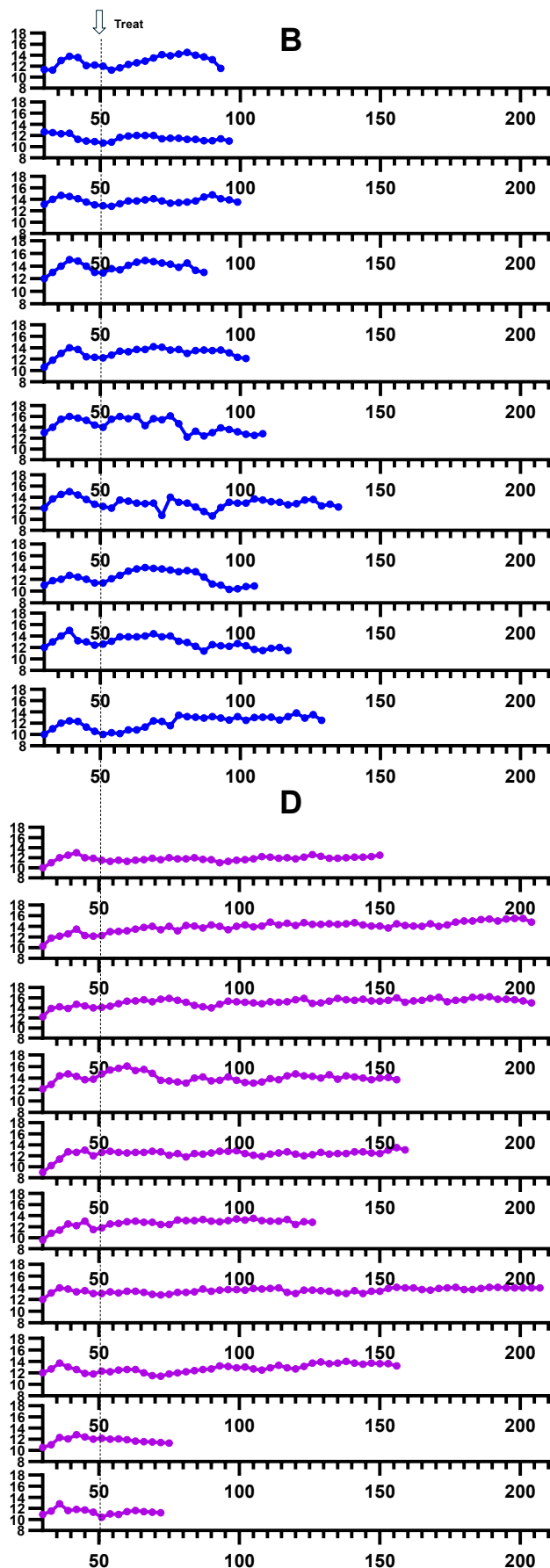

Days

Supplement: Supplement 1 [file media-1.pdf]
